# μ-1,2-Bis(diethyl­phosphino)ethane-κ^2^ *P*:*P*′-bis­{[1,2-bis­(diethyl­phosphino)ethane-κ^2^ *P*,*P*′]trichloridonitrosyl­tungsten(II)}

**DOI:** 10.1107/S1600536808001141

**Published:** 2008-01-16

**Authors:** Nataša Avramović, Olivier Blacque, Helmut W. Schmalle, Heinz Berke

**Affiliations:** a Anorganisch-Chemisches Institut der Universität Zürich, Winterthurerstrasse 190, CH-8057 Zürich, Switzerland

## Abstract

The title binuclear compound, [W_2_Cl_6_(NO)_2_(C_10_H_22_P_2_)_3_], contains two W atoms which are bridged by a bis­(diethyl­phosphino)­ethane (depe) ligand. The seven-coord­inated tungsten(II) centres display distorted penta­gonal–bipyramidal geometries with *trans* nitrosyl and chloride ligands. The title mol­ecule lies on a crystallographic inversion centre. The ethane group of the non-bridging depe ligand is positionally disordered, with site-occupancy factors of 0.63 and 0.37. In the crystal structure, the binuclear mol­ecules are linked by weak inter­molecular C—H⋯O and C—H⋯Cl inter­actions. In addition, weak intra­molecular C—H⋯Cl inter­actions are also present.

## Related literature

For related literature, see: Avramović *et al.* (2008 ▶); Bencze & Kohàn (1982 ▶); Campbell *et al.* (1985 ▶); Carmona *et al.* (1989 ▶); Desiraju & Steiner (1999 ▶); Han & Coucouvanis (2002 ▶); Hunter & Legzdins (1984 ▶); Landau *et al.* (1999 ▶); Zeng *et al.* (1994 ▶).
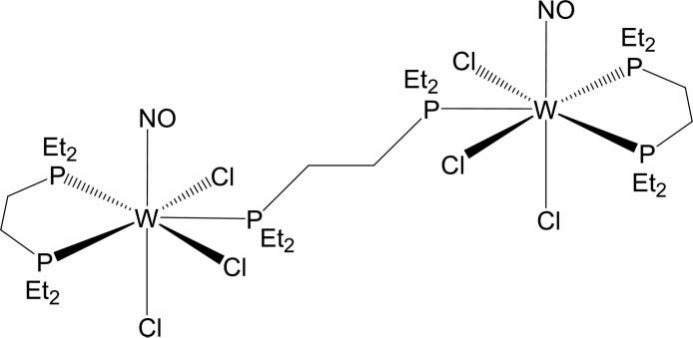


## Experimental

### 

#### Crystal data

[W_2_Cl_6_(NO)_2_(C_10_H_22_P_2_)_3_]
*M* *_r_* = 1259.10
Orthorhombic, 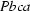

*a* = 12.6406 (14) Å
*b* = 17.6485 (14) Å
*c* = 20.5243 (17) Å
*V* = 4578.7 (7) Å^3^
*Z* = 4
Mo *K*α radiation
μ = 5.61 mm^−1^
*T* = 183 (2) K
0.26 × 0.20 × 0.15 mm

#### Data collection

Stoe IPDS diffractometer
Absorption correction: numerical (Coppens *et al.*, 1965 ▶) *T*_min_ = 0.329, *T*_max_ = 0.499
52250 measured reflections
3982 independent reflections
3026 reflections with *I* > 2σ(*I*)
*R*_int_ = 0.079

#### Refinement

*R*[*F* ^2^ > 2σ(*F* ^2^)] = 0.024
*wR*(*F* ^2^) = 0.056
*S* = 0.84
3982 reflections
224 parameters
1 restraint
H-atom parameters constrained
Δρ_max_ = 1.04 e Å^−3^
Δρ_min_ = −1.27 e Å^−3^

### 

Data collection: *IPDS Software* (Stoe & Cie, 1999 ▶); cell refinement: *IPDS Software*; data reduction: *X-RED* (Stoe & Cie, 1999 ▶); program(s) used to solve structure: *SHELXS97* (Sheldrick, 2008 ▶); program(s) used to refine structure: *SHELXL97* (Sheldrick, 2008 ▶); molecular graphics: *ORTEP-3 for Windows* (Farrugia, 1997 ▶); software used to prepare material for publication: *WinGX* (Farrugia, 1999 ▶), *SHELXL97* and *PLATON* (Spek, 2003 ▶).

## Supplementary Material

Crystal structure: contains datablocks global, I. DOI: 10.1107/S1600536808001141/su2041sup1.cif

Structure factors: contains datablocks I. DOI: 10.1107/S1600536808001141/su2041Isup2.hkl

Additional supplementary materials:  crystallographic information; 3D view; checkCIF report

## Figures and Tables

**Table:** 

W1—P1	2.5598 (13)
W1—P2	2.5675 (14)
W1—P3	2.6051 (12)
W1—Cl1	2.4750 (12)
W1—Cl2	2.4703 (11)
W1—Cl3	2.4905 (11)
W1—N1	1.783 (4)

**Table:** 

N1—W1—Cl1	98.06 (14)
N1—W1—Cl2	99.08 (13)
N1—W1—Cl3	177.72 (13)
Cl1—W1—Cl2	141.66 (4)
Cl2—W1—Cl3	82.27 (4)
Cl1—W1—Cl3	81.87 (4)
Cl1—W1—P2	71.30 (5)
Cl1—W1—P3	73.09 (4)
Cl2—W1—P1	70.77 (4)
Cl2—W1—P3	73.54 (4)
Cl3—W1—P1	90.21 (4)
P1—W1—P2	72.90 (5)

Symmetry code: (i) 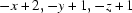.

**Table 2:** Hydrogen-bond geometry (Å, °)

*D*—H⋯*A*	*D*—H	H⋯*A*	*D*⋯*A*	*D*—H⋯*A*
C5—H5*B*⋯Cl2	0.97	2.81	3.196 (7)	105
C11—H11*A*⋯Cl1	0.97	2.71	3.129 (7)	107
C13—H13*B*⋯Cl1	0.97	2.75	3.132 (5)	104
C14—H14*A*⋯Cl2	0.97	2.76	3.150 (5)	105
C13—H13*A*⋯Cl1^i^	0.97	2.80	3.425 (5)	123
C10—H10*A*⋯Cl3^ii^	0.96	2.81	3.726 (7)	161
C2—H2*A*⋯O1^iii^	0.97	2.65	3.579 (10)	161
C4—H4*B*⋯O1^iii^	0.97	2.56	3.470 (15)	156

Symmetry codes: (i) 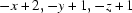; (ii) 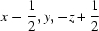; (iii) 
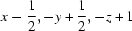
.

**Fig. 1.**
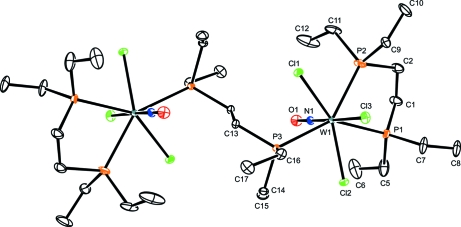
View of the molecular structure of the title compound, showing the atom-labeling scheme and displacement ellipsoids drawn at the 30% probability level. The disordered atoms, C3 and C4, and the hydrogen atoms have been omitted for clarity. Symmetry transformation: -x + 2, -y + 1, -z + 1.

**Fig. 2.**
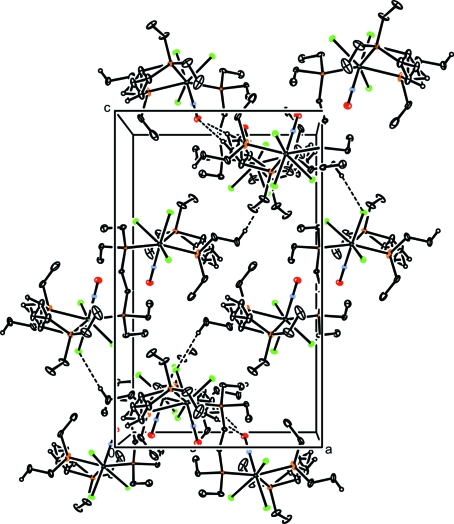
A view, along the b axis, of the crystal structure of the title compound. The intermolecular C—H···Cl and C—H···O hydrogen bonds are shown as dashed lines. Hydrogen atoms not involved in hydrogen bonding have been omitted for clarity.

## supplementary crystallographic information

### Comment

The mononuclear compound [W(Cl)_2_(NO)(dmpe)_2_](Cl) was previously obtained by the reaction of [W(Cl)_3_(NO)(NCCH_3_)_2_] with 2.5 equivalents of dmpe [1,2-bis(dimethylphosphino)ethane] at room temperature in tetrahydrofuran (Avramović *et al.*, 2008). The synthesis of the analogous compound [W(Cl)_2_(NO)(depe)_2_](Cl) was also attempted by the same procedure using the bidentate ligand depe [1,2-bis(diethylphosphino)ethane]. Presumably because of steric factors, only the binuclear compound [W(Cl)_3_(NO)(depe)]_2_(µ-depe) was formed instead of the expected mononuclear compound.

The title compound consists of two metal units bridged by a depe ligand (see: Campbell *et al.*, 1985; Han & Coucouvanis, 2002; Zeng *et al.*, 1994; Landau *et al.*, 1999). Both tungsten centers are crystallographically equivalent since the molecule lies on a crystallographic inversion center (Fig. 1). The geometry at tungsten(II) is very similar to that in [W(Cl)_2_(NO)(dmpe)_2_](Cl) with a distorted pentagonal bipyramidal coordination. A second chloride ligand takes the place of one phosphorus in [W(Cl)_2_(NO)(dmpe)_2_](Cl) to complete the equatorial plane of the polyhedron (P1, P2, P3, Cl1 and Cl2). The five equatorial bond angles, in the range 70.8 - 73.5°, are close to the theoretically average angle of 72°. It is worth noting that the five equatorial atoms are not completely coplanar. Atoms Cl1 and Cl2 deviate from the P1—P2—P3 plane, toward the third chloride atom Cl3, by 0.303 (2) and 0.543 (2) Å, respectively. Nevertheless, this geometry is clearly different to that observed for the related compound Mo(Cl)_3_(NO)(PMe_3_)_3_, for which the coordination polyhedron is described as a capped-octahedron (Carmona *et al.*, 1989).

All the chloride ligands are engaged in hydrogen bonding, with atoms Cl1 and Cl2 involved in ten intramolecular interactions with CH_2_ hydrogen atoms within the dimer, and atom Cl3 (*trans* to the nitrosyl group) in one weak intermolecular interaction with the methyl hydrogen atom H10A (Fig. 2, Table 1). The C10···Cl3 donor-acceptor distance of 3.726 (7) Å represents a rather weak interaction of this type (Desiraju & Steiner, 1999). In addition, the binuclear complexes are linked by two weak intermolecular C—H···O hydrogen bonds between the nitrosyl oxygen atom and the hydrogen atoms of the disordered ethane bridge.

### Experimental

[W(Cl)_3_(NO)(depe)]_2_(µ-depe) was prepared from the complex [W(Cl)_3_(NO)(CH_3_CN)_2_]. The latter is easily synthesized by the reaction of W(Cl)_6_ with NO gas in dichloromethane in the presence of acetonitrile at room temperature, according to a literature procedure (Bencze & Kohàn, 1982; Hunter & Legzdins, 1984). 5.00 g (12.6 mmol) of WCl_6_ and 1.32 ml (25.2 mmol) of acetonitrile were dissolved in 180 ml of dichloromethane in a 500 ml three-necked flask. Nitric oxide was passed through the solution, which was stirred at room temperature until the dark purple colour of the solution turned to the light green precipitate after *ca* 1 h. The volume of the final mixture was reduced to 50 ml *in vacuo* and the mixture was then cooled to 0°C for 15 min. The precipitate was isolated by filtration and the collected solid was then washed first with cold dichloromethane (2 *x* 10 ml at 0°C) and then with hexane (4 *x* 20 ml) at room temperature. Final drying of the solid under vacuum for 18 h afforded the yellow-green [W(Cl)_3_(NO)(CH_3_CN)_2_] compound. 0.200 g (0.497 mmol) of [W(Cl)_3_(NO)(NCCH_3_)_2_] was dissolved in 20 ml of tetrahydrofurane in a Young tap Schlenk and the depe ligand (0.29 ml, 1.243 mmol) was syringed into the solution. The solution was stirred at room temperature for 24 h, then filtered and the solvent removed under vacuum. The resulting solid was crystallized from dichloromethane at room temperature and gave light-green crystals of the title compound.

Yield: 0.266 g (85%).

IR (cm^-1^, CH_2_Cl_2_): 1520 (NO).

^1^H NMR (200.0 MHz, CD_2_Cl_2_, 25°C): 2.69 (m, 4H, P(C*H*_2_)_2_P); 2.39 (m, 8H, PC*H*_2_CH_3_); 2.19 (16*H*, PC*H*_2_CH_3_), 2.06 (m, 8H, P(C*H*_2_)_2_P), 1.25 (m, 24H, PCH_2_C*H*_3_), 1.19 (m,12*H*, PCH_2_C*H*_3_).

^31^P{^1^H} NMR (80.9 MHz, CD_2_Cl_2_, 25°C): 46.8 (m, P(CH_2_)_2_*P*), 45.8 (m, *P*(CH_2_)_2_P) and 21.4 (m, *P*(CH_2_)_2_*P*), ^2^J_PN_ = 15.6 Hz; ^1^J_PW_ = 203 Hz.

^13^C{^1^H} NMR (50.3 MHz, CD_2_Cl_2_, 25°C): 8.8 (m, PCH_2_*C*H_3_), 9.2 (s, PCH_2_*C*H_3_), 15.7 (m, P*C*H_2_CH_3_), 18.5 (m, P*C*H_2_CH_3_), 20.0 (m, P(*C*H_2_)_2_P).

Anal. Calcd for C_30_H_72_Cl_6_N_2_O_2_P_6_W_2_: C, 28.60; H, 5.72; N, 2.22. Found: C, 28.87; H, 6.01; N, 1.98.

### Refinement

The H atom were included in calculated positions and treated as riding atoms: C—H distances 0.96–0.99 Å and *U*_iso_(H) = 1.2 or 1.5*U*_eq_(C). The ethyl group of one depe ligand is positionally disordered; refined site occupancy factors 0.631 (8):0.369 (8).

### Crystal data

**Table:**

[W_2_Cl_6_(NO)_2_(C_10_H_22_P_2_)_3_]	*F*_000_ = 2488
*M**_r_* = 1259.10	*D*_x_ = 1.827 Mg m^−^^3^
Orthorhombic, *P**b**c**a*	Mo *K*α radiation λ = 0.71073 Å
Hall symbol: -P 2ac 2ab	Cell parameters from 7997 reflections
*a* = 12.6406 (14) Å	θ = 2.8–30.4º
*b* = 17.6485 (14) Å	µ = 5.61 mm^−^^1^
*c* = 20.5243 (17) Å	*T* = 183 (2) K
*V* = 4578.7 (7) Å^3^	Block, yellow
*Z* = 4	0.26 × 0.20 × 0.15 mm

### Data collection

**Table:**

Stoe IPDS diffractometer	3982 independent reflections
Radiation source: fine-focus sealed tube	3026 reflections with *I* > 2σ(*I*)
Monochromator: graphite	*R*_int_ = 0.079
*T* = 183(2) K	θ_max_ = 25.0º
φ oscillation scan	θ_min_ = 2.8º
Absorption correction: numerical(Coppens *et al.*, 1965)	*h* = −15→15
*T*_min_ = 0.329, *T*_max_ = 0.499	*k* = −20→20
52250 measured reflections	*l* = −24→24

### Refinement

**Table:**

Refinement on *F*^2^	Secondary atom site location: difference Fourier map
Least-squares matrix: full	Hydrogen site location: inferred from neighbouring sites
*R*[*F*^2^ > 2σ(*F*^2^)] = 0.024	H-atom parameters constrained
*wR*(*F*^2^) = 0.056	*w* = 1/[σ^2^(*F*_o_^2^) + (0.0357*P*)^2^] where *P* = (*F*_o_^2^ + 2*F*_c_^2^)/3
*S* = 0.85	(Δ/σ)_max_ = 0.001
3982 reflections	Δρ_max_ = 1.04 e Å^−^^3^
224 parameters	Δρ_min_ = −1.27 e Å^−^^3^
1 restraint	Extinction correction: none
Primary atom site location: structure-invariant direct methods	

### Special details

**Table:**

Geometry. All e.s.d.'s (except the e.s.d. in the dihedral angle between two l.s. planes) are estimated using the full covariance matrix. The cell e.s.d.'s are taken into account individually in the estimation of e.s.d.'s in distances, angles and torsion angles; correlations between e.s.d.'s in cell parameters are only used when they are defined by crystal symmetry. An approximate (isotropic) treatment of cell e.s.d.'s is used for estimating e.s.d.'s involving l.s. planes.
Refinement. Refinement of *F*^2^ against ALL reflections. The weighted *R*-factor *wR* and goodness of fit *S* are based on *F*^2^, conventional *R*-factors *R* are based on *F*, with *F* set to zero for negative *F*^2^. The threshold expression of *F*^2^ > σ(*F*^2^) is used only for calculating *R*-factors(gt) *etc*. and is not relevant to the choice of reflections for refinement. *R*-factors based on *F*^2^ are statistically about twice as large as those based on *F*, and *R*- factors based on ALL data will be even larger.

### Fractional atomic coordinates and isotropic or equivalent isotropic displacement parameters (Å^2^)

**Table:**

	*x*	*y*	*z*	*U*_iso_*/*U*_eq_	Occ. (<1)
W1	0.826046 (13)	0.388044 (11)	0.379416 (7)	0.01301 (6)	
Cl1	0.77419 (9)	0.50925 (7)	0.43099 (5)	0.0229 (3)	
Cl2	0.95733 (9)	0.33344 (8)	0.30362 (5)	0.0232 (3)	
Cl3	0.76818 (10)	0.45607 (8)	0.27925 (5)	0.0261 (3)	
P1	0.75410 (11)	0.26356 (8)	0.33323 (6)	0.0221 (3)	
P2	0.62919 (11)	0.38137 (9)	0.40971 (8)	0.0319 (3)	
P3	0.99811 (9)	0.46776 (7)	0.39288 (5)	0.0141 (3)	
N1	0.8622 (3)	0.3381 (2)	0.45154 (17)	0.0180 (9)	
O1	0.8860 (3)	0.3047 (2)	0.50083 (16)	0.0282 (9)	
C1	0.6454 (7)	0.2268 (5)	0.3833 (5)	0.0341 (16)	0.631 (8)
H1A	0.6141	0.1827	0.3627	0.041*	0.631 (8)
H1B	0.6716	0.2120	0.4259	0.041*	0.631 (8)
C2	0.5642 (6)	0.2887 (5)	0.3901 (5)	0.0341 (16)	0.631 (8)
H2A	0.5147	0.2756	0.4244	0.041*	0.631 (8)
H2B	0.5248	0.2934	0.3497	0.041*	0.631 (8)
C3	0.6069 (13)	0.2531 (10)	0.3577 (7)	0.0341 (16)	0.369 (8)
H3A	0.5619	0.2840	0.3301	0.041*	0.369 (8)
H3B	0.5842	0.2007	0.3547	0.041*	0.369 (8)
C4	0.6042 (13)	0.2805 (9)	0.4274 (7)	0.0341 (16)	0.369 (8)
H4A	0.6597	0.2580	0.4538	0.041*	0.369 (8)
H4B	0.5360	0.2723	0.4478	0.041*	0.369 (8)
C5	0.8399 (6)	0.1786 (4)	0.3396 (3)	0.0505 (18)	
H5A	0.7963	0.1345	0.3313	0.061*	
H5B	0.8917	0.1813	0.3048	0.061*	
C6	0.8966 (6)	0.1655 (4)	0.4000 (4)	0.072 (3)	
H6A	0.9530	0.2016	0.4040	0.107*	
H6B	0.9255	0.1152	0.3999	0.107*	
H6C	0.8489	0.1709	0.4361	0.107*	
C7	0.7194 (6)	0.2647 (3)	0.2484 (3)	0.0408 (15)	
H7A	0.6749	0.3085	0.2405	0.049*	
H7B	0.7837	0.2714	0.2232	0.049*	
C8	0.6617 (5)	0.1942 (4)	0.2226 (3)	0.0484 (18)	
H8A	0.7019	0.1497	0.2332	0.073*	
H8B	0.6542	0.1980	0.1762	0.073*	
H8C	0.5930	0.1909	0.2423	0.073*	
C9	0.5410 (4)	0.4353 (5)	0.3574 (3)	0.051 (2)	
H9A	0.5584	0.4886	0.3613	0.062*	
H9B	0.5535	0.4205	0.3126	0.062*	
C10	0.4233 (5)	0.4248 (7)	0.3728 (3)	0.099 (4)	
H10A	0.3817	0.4455	0.3380	0.148*	
H10B	0.4065	0.4504	0.4127	0.148*	
H10C	0.4080	0.3718	0.3773	0.148*	
C11	0.5917 (5)	0.4121 (4)	0.4913 (3)	0.0462 (19)	
H11A	0.5940	0.4670	0.4927	0.055*	
H11B	0.5192	0.3968	0.4992	0.055*	
C12	0.6603 (6)	0.3814 (6)	0.5455 (3)	0.082 (3)	
H12A	0.6658	0.3273	0.5415	0.123*	
H12B	0.6291	0.3938	0.5868	0.123*	
H12C	0.7295	0.4035	0.5428	0.123*	
C13	1.0135 (4)	0.5223 (3)	0.46896 (19)	0.0157 (10)	
H13A	1.0860	0.5398	0.4720	0.019*	
H13B	0.9682	0.5666	0.4668	0.019*	
C14	1.1275 (4)	0.4204 (3)	0.3871 (2)	0.0207 (10)	
H14A	1.1397	0.4068	0.3419	0.025*	
H14B	1.1818	0.4565	0.3994	0.025*	
C15	1.1407 (4)	0.3499 (3)	0.4285 (3)	0.0306 (13)	
H15A	1.1336	0.3630	0.4737	0.046*	
H15B	1.2094	0.3285	0.4211	0.046*	
H15C	1.0874	0.3135	0.4170	0.046*	
C16	1.0053 (4)	0.5396 (3)	0.3298 (2)	0.0239 (12)	
H16A	1.0112	0.5143	0.2880	0.029*	
H16B	0.9390	0.5674	0.3297	0.029*	
C17	1.0947 (4)	0.5963 (3)	0.3352 (3)	0.0340 (14)	
H17A	1.0875	0.6246	0.3749	0.051*	
H17B	1.0923	0.6305	0.2988	0.051*	
H17C	1.1612	0.5700	0.3352	0.051*	

### Atomic displacement parameters (Å^2^)

**Table:**

	*U*^11^	*U*^22^	*U*^33^	*U*^12^	*U*^13^	*U*^23^
W1	0.01297 (9)	0.01534 (10)	0.01072 (9)	0.00073 (8)	0.00014 (7)	0.00121 (8)
Cl1	0.0221 (6)	0.0214 (7)	0.0251 (6)	0.0079 (5)	−0.0026 (5)	−0.0062 (5)
Cl2	0.0198 (6)	0.0291 (7)	0.0206 (6)	0.0006 (5)	0.0059 (5)	−0.0074 (5)
Cl3	0.0311 (7)	0.0298 (8)	0.0174 (5)	0.0003 (6)	−0.0063 (5)	0.0073 (5)
P1	0.0230 (7)	0.0242 (8)	0.0190 (6)	−0.0073 (6)	0.0008 (5)	−0.0066 (5)
P2	0.0183 (6)	0.0265 (8)	0.0510 (9)	−0.0004 (7)	0.0133 (6)	−0.0013 (7)
P3	0.0145 (6)	0.0166 (6)	0.0113 (5)	−0.0011 (5)	0.0025 (4)	0.0009 (4)
N1	0.0156 (19)	0.025 (2)	0.0137 (18)	−0.0019 (18)	−0.0001 (15)	0.0010 (18)
O1	0.033 (2)	0.028 (2)	0.0232 (18)	0.0046 (17)	−0.0024 (16)	0.0112 (16)
C1	0.022 (4)	0.026 (4)	0.055 (5)	−0.006 (3)	0.011 (3)	0.000 (3)
C2	0.022 (4)	0.026 (4)	0.055 (5)	−0.006 (3)	0.011 (3)	0.000 (3)
C3	0.022 (4)	0.026 (4)	0.055 (5)	−0.006 (3)	0.011 (3)	0.000 (3)
C4	0.022 (4)	0.026 (4)	0.055 (5)	−0.006 (3)	0.011 (3)	0.000 (3)
C5	0.073 (5)	0.026 (4)	0.052 (4)	−0.012 (3)	−0.027 (4)	0.003 (3)
C6	0.068 (5)	0.020 (4)	0.127 (7)	0.004 (4)	−0.052 (5)	0.004 (4)
C7	0.065 (4)	0.026 (3)	0.032 (3)	−0.002 (3)	−0.029 (3)	−0.005 (3)
C8	0.063 (4)	0.035 (4)	0.047 (3)	0.010 (3)	−0.036 (3)	−0.019 (3)
C9	0.023 (3)	0.089 (6)	0.042 (3)	0.013 (3)	−0.013 (3)	−0.030 (4)
C10	0.015 (3)	0.233 (13)	0.049 (4)	0.007 (5)	−0.004 (3)	−0.047 (6)
C11	0.026 (3)	0.076 (6)	0.037 (3)	0.017 (3)	0.017 (3)	0.019 (3)
C12	0.045 (4)	0.143 (9)	0.058 (4)	0.040 (5)	0.025 (4)	0.062 (5)
C13	0.019 (2)	0.017 (3)	0.011 (2)	−0.006 (2)	0.0019 (18)	0.0006 (19)
C14	0.016 (2)	0.022 (3)	0.024 (2)	0.002 (2)	0.004 (2)	−0.002 (2)
C15	0.028 (3)	0.030 (3)	0.034 (3)	0.013 (2)	−0.001 (2)	0.000 (3)
C16	0.032 (3)	0.024 (3)	0.017 (2)	−0.004 (2)	0.004 (2)	0.006 (2)
C17	0.034 (3)	0.032 (4)	0.036 (3)	−0.008 (3)	0.003 (2)	0.017 (3)

### Geometric parameters (Å, °)

**Table:**

W1—P1	2.5598 (13)	C6—H6C	0.9600
W1—P2	2.5675 (14)	C7—C8	1.536 (8)
W1—P3	2.6051 (12)	C7—H7A	0.9700
W1—Cl1	2.4750 (12)	C7—H7B	0.9700
W1—Cl2	2.4703 (11)	C8—H8A	0.9600
W1—Cl3	2.4905 (11)	C8—H8B	0.9600
W1—N1	1.783 (4)	C8—H8C	0.9600
P1—C7	1.796 (5)	C9—C10	1.531 (8)
P1—C1	1.834 (8)	C9—H9A	0.9700
P1—C5	1.855 (7)	C9—H9B	0.9700
P1—C3	1.936 (15)	C10—H10A	0.9600
P2—C9	1.817 (7)	C10—H10B	0.9600
P2—C11	1.822 (6)	C10—H10C	0.9600
P2—C4	1.844 (16)	C11—C12	1.512 (8)
P2—C2	1.874 (9)	C11—H11A	0.9700
P3—C16	1.815 (5)	C11—H11B	0.9700
P3—C14	1.840 (5)	C12—H12A	0.9600
P3—C13	1.844 (4)	C12—H12B	0.9600
N1—O1	1.208 (5)	C12—H12C	0.9600
C1—C2	1.506 (10)	C13—C13^i^	1.535 (8)
C1—H1A	0.9700	C13—H13A	0.9700
C1—H1B	0.9700	C13—H13B	0.9700
C2—H2A	0.9700	C14—C15	1.516 (7)
C2—H2B	0.9700	C14—H14A	0.9700
C3—C4	1.511 (10)	C14—H14B	0.9700
C3—H3A	0.9700	C15—H15A	0.9600
C3—H3B	0.9700	C15—H15B	0.9600
C4—H4A	0.9700	C15—H15C	0.9600
C4—H4B	0.9700	C16—C17	1.514 (7)
C5—C6	1.450 (9)	C16—H16A	0.9700
C5—H5A	0.9700	C16—H16B	0.9700
C5—H5B	0.9700	C17—H17A	0.9600
C6—H6A	0.9600	C17—H17B	0.9600
C6—H6B	0.9600	C17—H17C	0.9600
			
N1—W1—Cl1	98.06 (14)	P1—C5—H5B	107.7
N1—W1—Cl2	99.08 (13)	H5A—C5—H5B	107.1
N1—W1—Cl3	177.72 (13)	C5—C6—H6A	109.5
Cl1—W1—Cl2	141.66 (4)	C5—C6—H6B	109.5
Cl2—W1—Cl3	82.27 (4)	H6A—C6—H6B	109.5
Cl1—W1—Cl3	81.87 (4)	C5—C6—H6C	109.5
N1—W1—P1	88.51 (14)	H6A—C6—H6C	109.5
Cl1—W1—P1	143.71 (4)	H6B—C6—H6C	109.5
Cl1—W1—P2	71.30 (5)	C8—C7—P1	116.2 (4)
Cl1—W1—P3	73.09 (4)	C8—C7—H7A	108.2
Cl2—W1—P1	70.77 (4)	P1—C7—H7A	108.2
Cl2—W1—P3	73.54 (4)	C8—C7—H7B	108.2
Cl3—W1—P1	90.21 (4)	P1—C7—H7B	108.2
N1—W1—P2	91.41 (13)	H7A—C7—H7B	107.4
Cl2—W1—P2	141.78 (5)	C7—C8—H8A	109.5
Cl3—W1—P2	86.41 (5)	C7—C8—H8B	109.5
P1—W1—P2	72.90 (5)	H8A—C8—H8B	109.5
N1—W1—P3	88.00 (13)	C7—C8—H8C	109.5
Cl3—W1—P3	94.16 (4)	H8A—C8—H8C	109.5
P1—W1—P3	143.07 (4)	H8B—C8—H8C	109.5
P2—W1—P3	143.92 (4)	C10—C9—P2	114.3 (6)
C7—P1—C1	111.4 (4)	C10—C9—H9A	108.7
C7—P1—C5	102.7 (3)	P2—C9—H9A	108.7
C1—P1—C5	96.5 (4)	C10—C9—H9B	108.7
C7—P1—C3	91.0 (5)	P2—C9—H9B	108.7
C5—P1—C3	117.8 (6)	H9A—C9—H9B	107.6
C7—P1—W1	115.9 (2)	C9—C10—H10A	109.5
C1—P1—W1	111.2 (3)	C9—C10—H10B	109.5
C5—P1—W1	117.3 (2)	H10A—C10—H10B	109.5
C3—P1—W1	109.1 (5)	C9—C10—H10C	109.5
C9—P2—C11	103.1 (3)	H10A—C10—H10C	109.5
C9—P2—C4	121.1 (6)	H10B—C10—H10C	109.5
C11—P2—C4	93.5 (5)	C12—C11—P2	114.9 (5)
C9—P2—C2	93.5 (4)	C12—C11—H11A	108.5
C11—P2—C2	110.1 (4)	P2—C11—H11A	108.5
C9—P2—W1	115.3 (2)	C12—C11—H11B	108.5
C11—P2—W1	117.4 (2)	P2—C11—H11B	108.5
C4—P2—W1	104.9 (5)	H11A—C11—H11B	107.5
C2—P2—W1	114.4 (3)	C11—C12—H12A	109.5
C16—P3—C14	103.1 (2)	C11—C12—H12B	109.5
C16—P3—C13	103.6 (2)	H12A—C12—H12B	109.5
C14—P3—C13	101.4 (2)	C11—C12—H12C	109.5
C16—P3—W1	110.08 (17)	H12A—C12—H12C	109.5
C14—P3—W1	119.35 (17)	H12B—C12—H12C	109.5
C13—P3—W1	117.34 (15)	C13^i^—C13—P3	114.4 (4)
O1—N1—W1	179.3 (4)	C13^i^—C13—H13A	108.7
C2—C1—P1	107.8 (7)	P3—C13—H13A	108.7
C2—C1—H1A	110.1	C13^i^—C13—H13B	108.7
P1—C1—H1A	110.1	P3—C13—H13B	108.7
C2—C1—H1B	110.1	H13A—C13—H13B	107.6
P1—C1—H1B	110.1	C15—C14—P3	115.7 (3)
H1A—C1—H1B	108.5	C15—C14—H14A	108.3
C1—C2—P2	110.7 (6)	P3—C14—H14A	108.3
C1—C2—H2A	109.5	C15—C14—H14B	108.3
P2—C2—H2A	109.5	P3—C14—H14B	108.3
C1—C2—H2B	109.5	H14A—C14—H14B	107.4
P2—C2—H2B	109.5	C14—C15—H15A	109.5
H2A—C2—H2B	108.1	C14—C15—H15B	109.5
C4—C3—P1	103.7 (10)	H15A—C15—H15B	109.5
C4—C3—H3A	111.0	C14—C15—H15C	109.5
P1—C3—H3A	111.0	H15A—C15—H15C	109.5
C4—C3—H3B	111.0	H15B—C15—H15C	109.5
P1—C3—H3B	111.0	C17—C16—P3	116.6 (4)
H3A—C3—H3B	109.0	C17—C16—H16A	108.2
C3—C4—P2	96.8 (11)	P3—C16—H16A	108.2
C3—C4—H4A	112.4	C17—C16—H16B	108.2
P2—C4—H4A	112.4	P3—C16—H16B	108.2
C3—C4—H4B	112.4	H16A—C16—H16B	107.3
P2—C4—H4B	112.4	C16—C17—H17A	109.5
H4A—C4—H4B	110.0	C16—C17—H17B	109.5
C6—C5—P1	118.6 (5)	H17A—C17—H17B	109.5
C6—C5—H5A	107.7	C16—C17—H17C	109.5
P1—C5—H5A	107.7	H17A—C17—H17C	109.5
C6—C5—H5B	107.7	H17B—C17—H17C	109.5
			
N1—W1—P1—C7	−169.6 (3)	Cl2—W1—P3—C14	−36.06 (18)
Cl2—W1—P1—C7	−69.4 (3)	Cl1—W1—P3—C14	163.07 (18)
Cl1—W1—P1—C7	88.8 (3)	Cl3—W1—P3—C14	−116.74 (18)
Cl3—W1—P1—C7	12.3 (3)	P1—W1—P3—C14	−20.84 (19)
P2—W1—P1—C7	98.5 (3)	P2—W1—P3—C14	153.66 (18)
P3—W1—P1—C7	−84.9 (3)	N1—W1—P3—C13	−59.1 (2)
N1—W1—P1—C1	61.9 (4)	Cl2—W1—P3—C13	−159.16 (18)
Cl2—W1—P1—C1	162.1 (4)	Cl1—W1—P3—C13	39.97 (17)
Cl1—W1—P1—C1	−39.7 (4)	Cl3—W1—P3—C13	120.16 (18)
Cl3—W1—P1—C1	−116.2 (4)	P1—W1—P3—C13	−143.94 (18)
P2—W1—P1—C1	−30.0 (4)	P2—W1—P3—C13	30.6 (2)
P3—W1—P1—C1	146.6 (4)	C7—P1—C1—C2	−77.2 (8)
N1—W1—P1—C5	−47.8 (3)	C5—P1—C1—C2	176.4 (7)
Cl2—W1—P1—C5	52.4 (3)	C3—P1—C1—C2	−36.6 (10)
Cl1—W1—P1—C5	−149.4 (3)	W1—P1—C1—C2	53.7 (8)
Cl3—W1—P1—C5	134.1 (3)	P1—C1—C2—P2	−45.4 (9)
P2—W1—P1—C5	−139.7 (3)	C9—P2—C2—C1	140.7 (8)
P3—W1—P1—C5	36.9 (3)	C11—P2—C2—C1	−113.9 (7)
N1—W1—P1—C3	89.5 (5)	C4—P2—C2—C1	−55.6 (10)
Cl2—W1—P1—C3	−170.3 (5)	W1—P2—C2—C1	20.8 (9)
Cl1—W1—P1—C3	−12.1 (5)	C7—P1—C3—C4	−158.7 (11)
Cl3—W1—P1—C3	−88.6 (5)	C1—P1—C3—C4	58.6 (11)
P2—W1—P1—C3	−2.4 (5)	C5—P1—C3—C4	96.3 (12)
P3—W1—P1—C3	174.2 (5)	W1—P1—C3—C4	−40.8 (13)
N1—W1—P2—C9	172.8 (3)	P1—C3—C4—P2	69.6 (11)
Cl2—W1—P2—C9	−80.6 (3)	C9—P2—C4—C3	58.9 (12)
Cl1—W1—P2—C9	74.7 (3)	C11—P2—C4—C3	166.5 (10)
Cl3—W1—P2—C9	−7.9 (3)	C2—P2—C4—C3	39.8 (8)
P1—W1—P2—C9	−99.2 (3)	W1—P2—C4—C3	−73.8 (11)
P3—W1—P2—C9	84.2 (3)	C7—P1—C5—C6	170.9 (6)
N1—W1—P2—C11	50.8 (3)	C1—P1—C5—C6	−75.4 (7)
Cl2—W1—P2—C11	157.5 (3)	C3—P1—C5—C6	−91.1 (8)
Cl1—W1—P2—C11	−47.2 (3)	W1—P1—C5—C6	42.5 (7)
Cl3—W1—P2—C11	−129.8 (3)	C1—P1—C7—C8	−43.2 (6)
P1—W1—P2—C11	138.8 (3)	C5—P1—C7—C8	59.1 (6)
P3—W1—P2—C11	−37.7 (3)	C3—P1—C7—C8	−59.7 (7)
N1—W1—P2—C4	−51.3 (5)	W1—P1—C7—C8	−171.6 (4)
Cl2—W1—P2—C4	55.3 (5)	C11—P2—C9—C10	−55.8 (6)
Cl1—W1—P2—C4	−149.4 (5)	C4—P2—C9—C10	46.6 (8)
Cl3—W1—P2—C4	128.0 (5)	C2—P2—C9—C10	55.7 (6)
P1—W1—P2—C4	36.7 (5)	W1—P2—C9—C10	174.8 (5)
P3—W1—P2—C4	−139.9 (5)	C9—P2—C11—C12	−174.5 (6)
N1—W1—P2—C2	−80.5 (4)	C4—P2—C11—C12	62.4 (8)
Cl2—W1—P2—C2	26.2 (4)	C2—P2—C11—C12	86.8 (7)
Cl1—W1—P2—C2	−178.5 (4)	W1—P2—C11—C12	−46.4 (7)
Cl3—W1—P2—C2	98.9 (4)	C16—P3—C13—C13^i^	167.4 (5)
P1—W1—P2—C2	7.5 (4)	C14—P3—C13—C13^i^	−85.9 (5)
P3—W1—P2—C2	−169.0 (4)	W1—P3—C13—C13^i^	45.9 (5)
N1—W1—P3—C16	−177.1 (2)	C16—P3—C14—C15	−172.3 (4)
Cl2—W1—P3—C16	82.81 (18)	C13—P3—C14—C15	80.6 (4)
Cl1—W1—P3—C16	−78.06 (18)	W1—P3—C14—C15	−50.0 (4)
Cl3—W1—P3—C16	2.13 (18)	C14—P3—C16—C17	−58.1 (5)
P1—W1—P3—C16	98.03 (19)	C13—P3—C16—C17	47.3 (5)
P2—W1—P3—C16	−87.5 (2)	W1—P3—C16—C17	173.5 (4)
N1—W1—P3—C14	64.0 (2)		

Symmetry codes: (i) −*x*+2, −*y*+1, −*z*+1.

### Hydrogen-bond geometry (Å, °)

**Table:**

*D*—H···*A*	*D*—H	H···*A*	*D*···*A*	*D*—H···*A*
C5—H5B···Cl2	0.97	2.81	3.196 (7)	105
C11—H11A···Cl1	0.97	2.71	3.129 (7)	107
C13—H13B···Cl1	0.97	2.75	3.132 (5)	104
C14—H14A···Cl2	0.97	2.76	3.150 (5)	105
C13—H13A···Cl1^i^	0.97	2.80	3.425 (5)	123
C10—H10A···Cl3^ii^	0.96	2.81	3.726 (7)	161
C2—H2A···O1^iii^	0.97	2.65	3.579 (10)	161
C4—H4B···O1^iii^	0.97	2.56	3.470 (15)	156

Symmetry codes: (i) −*x*+2, −*y*+1, −*z*+1; (ii) *x*−1/2, *y*, −*z*+1/2; (iii) *x*−1/2, −*y*+1/2, −*z*+1.

## References

[bb1] Avramović, N., Blacque, O., Schmalle, H. W. & Berke, H. (2008). *Acta Cryst.* E**64**, m245. 10.1107/S1600536807067128 PMC2915163 21200586

[bb2] Bencze, L. & Kohàn, J. (1982). *Inorg. Chim. Acta*, **65**, L17–L19.

[bb3] Campbell, F. L. III, Cotton, F. A. & Powell, G. L. (1985). *Inorg. Chem.* **24**, 4384–4389.

[bb4] Carmona, E., Gutiérrez-Puebla, E., Monge, A., Pérez, P. J. & Sanchez, L. J. (1989). *Inorg. Chem.* **28**, 2120–2127.

[bb5] Coppens, P., Leiserowitz, L. & Rabinovich, D. (1965). *Acta Cryst.* **18**, 1035–1038.

[bb6] Desiraju, G. R. & Steiner, T. (1999). *The Weak Hydrogen Bond*, pp. 215–221. Oxford University Press.

[bb7] Farrugia, L. J. (1997). *J. Appl. Cryst.* **30**, 565.

[bb8] Farrugia, L. J. (1999). *J. Appl. Cryst.* **32**, 837–838.

[bb9] Han, J. & Coucouvanis, D. (2002). *Inorg. Chem.* **41**, 2738–2746. 10.1021/ic010913+ 12005498

[bb10] Hunter, A. D. & Legzdins, P. (1984). *Inorg. Chem.* **23**, 4198–4204.

[bb11] Landau, S. E., Morris, R. H. & Lough, A. J. (1999). *Inorg. Chem.* **38**, 6060–6068. 10.1021/ic990876a 11671314

[bb12] Sheldrick, G. M. (2008). *Acta Cryst.* A**64**, 112–122. 10.1107/S0108767307043930 18156677

[bb13] Spek, A. L. (2003). *J. Appl. Cryst.* **36**, 7–13.

[bb14] Stoe & Cie (1999). *IPDS Software and *X-RED** Stoe & Cie, Darmstadt, Germany.

[bb15] Zeng, D., Hampden-Smith, M. J. & Larson, E. M. (1994). *Acta Cryst.* C**50**, 1000–1002.

